# Exploring the interplay of depression, sleep quality, and hearing in tinnitus-related handicap: insights from polysomnography and pure-tone audiometry

**DOI:** 10.1186/s12888-024-05912-y

**Published:** 2024-06-19

**Authors:** Ting-Gang Chang, Yi-Ting Yao, Chiann-Yi Hsu, Ting-Ting Yen

**Affiliations:** 1 Department of Psychiatry, Taichung Veterans General Hospital, Taichung, Taiwan; 2 Department of Otorhinolaryngology, Taichung Veterans General Hospital, Taichung, Taiwan; 3 Biostatistics Task Force of Taichung Veterans General Hospital, Taichung, Taiwan; 4 School of Medicine, National Yang Ming Chiao Tung University, Taipei, Taiwan

**Keywords:** Depression, Polysomnography, Pure-tone audiometry, Sleep quality, Tinnitus

## Abstract

**Background:**

Tinnitus affects approximately 740 million adults globally, involving hearing, emotion, and sleep systems. However, studies using polysomnography and pure-tone audiometry (PTA) are limited. We aimed to assess the correlation between tinnitus and hearing, sleep quality, characteristics, and depression using polysomnography and PTA.

**Methods:**

In this cross-sectional study, we divided participants into tinnitus and non-tinnitus groups. We included 100 outpatients (65 with tinnitus, 35 without) from a medical center in Taiwan, who underwent polysomnography and completed rating scales including the Patient Health Questionnaire-9 (PHQ-9), Chinese version of the Pittsburgh Sleep Quality Index (PSQI), and Chinese-Mandarin version of the Tinnitus Handicap Inventory (THI-CM). We analyzed correlations, conducted group comparisons, assessed factors related to THI-CM scores, constructed ROC curves to predict depression in the tinnitus group, and performed multinomial and logistic regression to explore associations.

**Results:**

Descriptive statistics identified a cohort with mean age 53.9 ± 12.80 years, 63% exhibited PHQ-9 scores ≥ 10, and 66% had Apnea–Hypopnea Index (AHI) > 5. The ratio of rapid eye movement and deep sleep to stage 1 + 2 sleep was relatively low and non-significant. Likewise, leg movements was higher in the tinnitus group but not statistically significant. In the tinnitus group, 63.08% had depression, and 81.54% had AHI > 5. Univariate logistic regression linked tinnitus to AHI > 5 (Odds ratio (OR) 2.67, *p* = 0.026) and male sex (OR 2.49, *p* = 0.034). A moderate positive correlation was found between the THI-CM score and PHQ-9 score (rs = 0.50, *p* < 0.001). Further adjustment for obstructive sleep apnea showed associations between PHQ-9 (total score) or depression and THI-CM Grade 3–5 (OR = 1.28; OR = 8.68). Single- and multifactor regression analyses highlighted significant associations of PSQI scores > 13 (OR 7.06, *p* = 0.018) and THI-CM scores > 47 (OR 7.43, *p* = 0.002) with depression.

**Conclusions:**

Our study recruited tinnitus participants with slight or mild hearing loss and mild tinnitus handicap. Depression was identified as a predominant factor in tinnitus-related handicap. The mild tinnitus handicap in tinnitus participants may explain the lack of significant differences in depression, sleep quality, and polysomnographic sleep characteristics between tinnitus and non-tinnitus groups. Further extensive and prospective studies are needed to elucidate the complex links among depression, sleep, and tinnitus.

**Supplementary Information:**

The online version contains supplementary material available at 10.1186/s12888-024-05912-y.

## Background

Tinnitus affects > 740 million adults worldwide, with > 120 million identifying it as a major problem, most of whom are aged ≥ 65 years [1]. Although there is no globally accepted classification of tinnitus, it can be divided into subjective, objective, primary, and secondary tinnitus [2]. The formation of tinnitus is believed to be related to the hearing, attention, memory, and emotional systems, but the relationship between them is complex [3, 4].

Emotional status, including depression and insomnia, is a key factor in the induction and exacerbation of tinnitus handicap [4, 5]. In the 2017 National Health Survey of 21 million patients with tinnitus (Integrated Health Interview Series tinnitus module), 25.6% reported experiencing depression in the previous 12 months, whereas this figure was only 9.1% in individuals without tinnitus [6]. A large contemporary comprehensive review suggests a 33% prevalence of depression among patients with tinnitus [7]. Additionally, the severity of tinnitus has been positively correlated with depression [6, 8].

Multiple studies have objectively analyzed the sleep of tinnitus patients through polysomnography (PSG) [9–13]. In their 2011 study, Hebert et al. found that while the tinnitus group exhibited poorer subjective sleep quality, there were no significant differences in objective polysomnographic sleep parameters compared to controls [9]. On the other hand, both Attanasio et al. and Teixeira et al. documented a deficit in both deep sleep and rapid eye movement (REM) sleep across all tinnitus patients [10, 11]. . A recent study revealed that individuals with Intermittent tinnitus during sleep exhibited significantly decreased deep sleep and REM sleep. The duration of REM sleep may correlate with overnight modulation of tinnitus [12]. Gao et al. utilized a meta-analysis approach to include eight case-control or cross-sectional studies and found that severe sleep apnea is significantly associated with a higher prevalence of tinnitus [14].

Fernandes et al. discovered that in females, the concurrent occurrence of painful temporomandibular Disorders and sleep bruxism is associated with an increased severity of tinnitus [15]. Camparis et al. found that the frequency of tinnitus is correlated with sleep bruxism [16]. A review of clinical and overnight polysomnographic data of 2,849 adults revealed that the severity of periodic limb movement syndrome did not significantly differ between subjects with and without tinnitus [17]. Tinnitus-related emotional, cognitive distress, and somatic complaints are correlated with insomnia severity [18]. Tinnitus severity is positively correlated with sleep quality before tinnitus onset, indicating that past sleep quality may affect the occurrence of tinnitus [19].

Furthermore, pure-tone audiometry (PTA) or other objective assessment tools are required to assess hearing thresholds in patients with tinnitus. Short-term stress can induce neuroplasticity, whereas long-term stress can cause hearing loss and increase tinnitus risk [4]. Stress may activate the inner ear’s local hypothalamic-pituitary-adrenal axis, impacting cochlear function and potentially leading to tinnitus. This process could involve changes in mineralocorticoid receptor function, potassium secretion, and neuronal plasticity within the auditory system [20]The relationship between depression, hearing, and tinnitus remains to be clarified.

A systematic review assessing the correlation between chronic tinnitus distress and symptoms of depression found selection bias in most included studies, as most participants were patients attending tinnitus clinics for medical treatment; additionally, the patients did not receive objective hearing tests [21]. Moreover, previous studies have examined the correlation between tinnitus and sleep characteristics measured using polysomnography, sleep quality measured using the Pittsburgh Sleep Quality Index (PSQI), and depression measured using the Patient Health Questionnaire-9 (PHQ-9).

Therefore, here, we invited patients with tinnitus (and controls) from otolaryngology and psychiatry units to increase patient diversity. We used polysomnography and PTA to explore the association between tinnitus and polysomnographic findings and hearing. Next, we verified the correlation between tinnitus, sleep quality, and depression. Based on the multifactorial nature of tinnitus involving hearing and emotional systems and associated sleep characteristics [3, 4], we hypothesized an association between tinnitus and severe depression, poor sleep quality, poor hearing, and sleep-related breathing problems.

## Methods

### Study setting, participants, and data

In this cross-sectional study, participants were divided into either a tinnitus or non-tinnitus group based on inclusion and exclusion criteria. All participants completed a one-time assessment, and the tinnitus group also underwent PTA to verify the correlation between tinnitus and depression, sleep quality, polysomnographic findings, and hearing.

Based on previous research, Hebert et al. recruited 22 participants with tinnitus and 22 without [9], while Teixeira et al. recruited 25 participants with tinnitus and 25 without [10]. Our study aimed to recruit 65 participants for both the tinnitus and non-tinnitus groups, but due to scheduling and budget constraints, we ultimately recruited 65 participants for the tinnitus group and 35 for the non-tinnitus group.

This study was conducted between January 2021 and July 2022 at Taichung Veterans General Hospital, a medical centre in Taiwan. The participants in the experimental and control groups were recruited from outpatient psychiatric and otolaryngology clinics. The inclusion criteria were: (1) chronic and subjective tinnitus lasting > 3 months; (2) age > 20 years; and (3) willingness to complete the tests and questionnaires. The exclusion criteria were: (1) sudden hearing loss; (2) Meniere’s disease; (3) organic brain disorders (e.g. acoustic neuroma, stroke); and (4) severe mental illness (e.g. schizophrenia, bipolar disorder, major depressive disorder). Participants who, upon physician inquiry, denied experiencing tinnitus or hearing issues were categorized within the control group.

We collected data on age, sex, body mass index (BMI), and neck and waist circumference. All participants underwent polysomnography and a rating scale study, including the PHQ-9, Chinese version of the PSQI, and Chinese-Mandarin version of the Tinnitus Handicap Inventory (THI-CM). Participants in the experimental group underwent additional PTA.

### Measurement methods

#### Full-night diagnostic polysomnography

All participants underwent a comprehensive diagnostic overnight sleep study using a standard system (Compumedics, E-series, Victoria, Australia). The recordings included: electroencephalography with electrodes placed at positions C3/A2, C4/A1, O1/A2, and O2/A1; electrooculography; submental and tibial electromyography; electrocardiography; oronasal airflow measured using a thermocouple; respiratory effort measured using inductive plethysmography, with one thoracic and one abdominal belt; arterial oxyhaemoglobin saturation measured using pulse oximetry; and snoring detected through a small microphone attached around the cricoid cartilage.

The arousal index was calculated by dividing the total number of arousal events by sleep duration in hours. Similarly, the snore index was determined by dividing the total number of snores by the sleep duration. The apnoea index was calculated as the total number of apnoea events divided by the total sleep time in hours, whereas the apnoea–hypopnoea index (AHI) was determined by dividing the total number of apnoea and hypopnoea events by the total sleep time. The severity of obstructive sleep apnoea syndrome (OSAS) was classified according to the AHI: ‘mild’, 5–14; ‘moderate’, 15–30; and ‘severe’, > 30 events per hour.

#### Pure-tone audiometry

Audiometry was performed using a pure-tone audiometer (Grason Stadler; Otometrics, USA). Pure-tone air and bone conduction hearing thresholds were evaluated using standard procedures at frequencies of 250, 500, 1000, 2000, 4000, and 8000 Hz. Pure-tone averages were calculated at frequencies of 500, 1000, 2000, and 4000 Hz. All decibel (dB) values in this study were related to dB hearing levels (dB HL). This study follows the classification of hearing loss established by The American Speech-Language-Hearing Association, which utilizes pure-tone audiometry. The classifications encompass normal hearing (-10 to 15 dB HL), slight hearing loss (16 to 25 dB HL), mild hearing loss (26 to 40 dB HL), moderate hearing loss (41 to 55 dB HL), moderately severe hearing loss (56 to 70 dB HL), severe hearing loss (71 to 90 dB HL), and profound hearing loss (91 dB HL and above) [22].

#### PHQ-9

The PHQ-9 is a nine-item instrument administered to patients in primary care settings to detect the presence and severity of depression. Each question has four options with a score of 0 to 3 points, and the total score for the nine questions is calculated: 10–14 represents mild depression; 15–19, moderate depression; and ≥ 20, severe depression. This can be used to establish a diagnosis of depression according to the DSM-5 criteria. Reliability was assessed by computing the correlation (0.84) between PHQ-9 scores obtained from in-person and telephonic interviews with the same patients [23]. In an assessment of construct validity, the correlation between the mental health scale scores on the PHQ-9 and 20-item Short Form Survey was 0.73. To evaluate the validity of the criteria, a mental health professional validated depression diagnoses using PHQ-9 scores from 580 participants, resulting in 88% sensitivity and 88% specificity [23].

#### Chinese version of the PSQI

The PSQI evaluates subjective sleep quality. It comprises 19 items that assess sleep patterns and quality over the previous month. The 19 items comprise seven dimensions: sleep quality, habitual sleep efficiency, sleep latency, daytime dysfunction, sleep duration, sleep disturbances, and the use of sleep medications. The sum of the responses to these seven components constitutes a global score ranging from 0 to 21, with lower scores indicating better sleep quality. Scores > 5 indicate possible sleep pathology [24]. The PSQI has been amended and translated, becoming a reliable and valid tool for detecting sleep pathology [25]. It is a useful, self-administered tool with high sensitivity. PSQI scores > 5 have yielded 98% sensitivity and 55% specificity.

#### Chinese-Mandarin version of the tinnitus handicap inventory

The THI comprises 25 items grouped into three subscales: functional (11 items), emotional (9 items), and catastrophic (5 items). There are three response options: no such phenomenon, mild, and severe. There are five grades of tinnitus handicap according to the score. Grade one (0–16 points) and grade two (18–36 points) indicates no or slight handicap and mild handicap, respectively. Individuals with hearing loss receive hearing aids and those without hearing loss are only monitored. Those with grade three (38–56 points), indicating moderate hearing loss, require evaluation and treatment for other items of tinnitus, and those with grade four (58–76 points) to five (78–100 points) require a complete tinnitus evaluation comprising medical and psychological examinations, as these indicate severe and catastrophic handicap, respectively. The original version of the THI was translated into Mandarin Chinese using the translation/back-translation method [26].

### Statistical analyses

Data were summarized using descriptive statistics. Correlations between tinnitus and related factors were assessed. Group comparisons were conducted using Chi-square, Fisher’s exact, Mann–Whitney, Kruskal-Wallis, and Dunn-Bonferroni post hoc tests. Spearman’s rank correlation coefficient was utilized to examine continuous factors associated with THI-CM scores. A receiver operating characteristic curve was plotted to predict depression using PSQI and THI-CM scores in the tinnitus group. Multinomial logistic regression was applied to explore associations between categorical THI-CM and other factors, while logistic regression was used to assess the relationship between depression and variables such as age, sex, PSQI, and THI-CM. A p-value of < 0.05 was considered statistically significant. SPSS Statistics, Version 22.0, was used for all analyses(IBM Corp., New York, USA).

## Results

### Participant characteristics

A total of 100 patients were enrolled: 65 in the tinnitus group and 35 in the non-tinnitus group. Table 1 presents their descriptive statistics. Among the 100 participants, there were 40 women and 60 men, with a mean age of 53.9 ± 12.80 years, BMI of 25.60 ± 4.02 kg/m^2^, and sleep AHI of 18.2 ± 19.67; additionally, 63% had a PHQ score ≥ 10 and 66% had an AHI > 5. Among the participants with tinnitus, 63.08% had depression and 81.54% had obstructive sleep apnoea. The average neck and waist circumferences were 36.6 ± 5.06 cm and 88.14 ± 11.19 cm, respectively.

**Table 1:** The characteristics and assessments results of all the participants (*n* = 100)

Characteristic	Total (*n* = 100)		Tinnitus				*p* value
			No (*n* = 35)		Yes (*n* = 65)		
Age	53.95	± 12.80	51.76	± 16.56	55.13	± 10.19	0.477
Sex							0.032*
Female count (sex ratio)	40	(40.00%)	19	(54.29%)	21	(32.31%)	
Male count (sex ratio)	60	(60.00%)	16	(45.71%)	44	(67.69%)	
PSQI (total score)	10.13	± 4.39	9.54	± 4.13	10.44	± 4.53	0.254
PSQI > 5(yes)	84	(84.00%)	31	(88.57%)	53	(81.54%)	0.360
Subjective sleep quality	1.72	± 0.81	1.54	± 0.82	1.82	± 0.79	0.101
Sleep latency	1.48	± 1.02	1.51	± 1.12	1.46	± 0.97	0.837
Sleep duration	1.64	± 0.92	1.51	± 0.89	1.71	± 0.93	0.261
Habitual sleep efficiency	0.95	± 1.11	1.00	± 1.14	0.92	± 1.11	0.756
Sleep disturbances	1.58	± 0.64	1.43	± 0.61	1.66	± 0.64	0.125
Use of sleep medication	1.59	± 1.41	1.51	± 1.38	1.63	± 1.43	0.788
Daytime dysfunction	1.16	± 0.94	1.03	± 0.89	1.23	± 0.96	0.355
PHQ-9 (total score)	7.26	± 5.28	7.69	± 6.47	7.03	± 4.55	0.997
PHQ-9 ≥ 10	63	(63.00%)	22	(62.86%)	41	(63.08%)	0.983
Severity of depression							0.376
Normal	37	(37.00%)	13	(37.14%)	24	(36.92%)	
Mild	34	(34.00%)	10	(28.57%)	24	(36.92%)	
Moderate	19	(19.00%)	7	(20.00%)	12	(18.46%)	
Moderate severe	8	(8.00%)	3	(8.57%)	5	(7.69%)	
Severe	2	(2.00%)	2	(5.71%)	0	(0.00%)	
Polysomnography							
Total recorded time	366.74	± 15.42	366.97	± 16.23	366.62	± 15.10	0.831
Total sleep time	308.31	± 40.68	301.09	± 41.54	312.20	± 40.00	0.129
Episode of apnoea	32.33	± 48.86	33.94	± 59.20	31.46	± 42.76	0.347
Obstructive apnoea	23.32	± 37.30	21.89	± 40.56	24.09	± 35.72	0.384
Central apnoea	6.57	± 15.28	7.74	± 15.92	5.94	± 15.01	0.879
Mix apnoea	2.44	± 8.11	4.31	± 12.75	1.43	± 3.54	0.750
Episode of hypopnoea	63.08	± 79.26	54.81	± 94.33	67.54	± 70.25	0.074
Longest period of apnoea	31.57	± 20.38	30.44	± 22.33	32.18	± 19.40	0.463
Lowest oxygen saturation	81.37	± 12.71	81.60	± 17.51	81.25	± 9.34	0.104
Apnoea index	6.26	± 9.24	6.34	± 10.69	6.21	± 8.44	0.445
AHI	18.29	± 19.67	16.11	± 21.81	19.47	± 18.49	0.099
AHI > 5 (Yes)	66	(66.00%)	18	(51.43%)	48	(73.85%)	0.024*
Severity of OSAS							0.057
Normal	34	(34.00%)	17	(48.57%)	17	(26.15%)	
Mild	27	(27.00%)	9	(25.71%)	18	(27.69%)	
Moderate	17	(17.00%)	2	(5.71%)	15	(23.08%)	
Severe	22	(22.00%)	7	(20.00%)	15	(23.08%)	
Snoring index	216.77	± 189.75	193.20	± 177.23	229.45	± 196.32	0.340
Arousal index	16.46	± 15.15	17.76	± 19.37	15.76	± 12.42	0.461
Leg movement index	18.93	± 29.05	13.07	± 20.77	22.09	± 32.36	0.143
Sleep efficiency	84.12	± 10.91	82.11	± 11.43	85.20	± 10.55	0.167
Stage N1	14.92	± 11.94	12.73	± 10.12	16.09	± 12.73	0.114
Stage N2	64.27	± 13.84	64.64	± 13.14	64.07	± 14.30	0.931
Stage N3	8.22	± 9.61	9.88	± 9.71	7.32	± 9.51	0.166
REM	12.59	± 6.52	12.74	± 6.26	12.52	± 6.70	0.756
BMI (kg/m^2^)	25.60	± 4.02	25.01	± 3.61	25.91	± 4.21	0.285
BMI classification							0.715
Normal	44	(44.44%)	17	(50.00%)	27	(41.54%)	
Overweight	25	(25.25%)	8	(23.53%)	17	(26.15%)	
Obesity	30	(30.30%)	9	(26.47%)	21	(32.31%)	
Neck circumference (cm)	36.60	± 5.06	35.84	± 4.21	37.03	± 5.47	0.421
Waist circumference (cm)	88.14	± 11.19	86.90	± 10.23	88.83	± 11.72	0.598

The Mann–Whitney U test and chi-square test were used to obtain the data. *p < 0.05, **p < 0.01. Continuous data are expressed as mean ± SD. Categorical data are expressed as number and percentage. PSQI, Pittsburgh Sleep Quality Index; PHQ-9, Patient Health Questionnaire-9; AHI, Apnoea–hypopnoea index; BMI, body mass index; REM, Rapid eye movement

Significant differences between the two groups were observed solely in the proportion of male participants (tinnitus group: 67.69%, non-tinnitus group: 45.71%) and in the percentage of those with an AHI greater than 5 (tinnitus group: 51.43%, non-tinnitus group: 73.85%). The severity of obstructive sleep apnea showed no significant difference between the two groups. In the tinnitus group, deep sleep (stage 3) was slightly lower at 7.32 ± 9.51, and REM sleep was also slightly lower at 12.52 ± 6.70 compared to the non-tinnitus group, but the differences were not significant. Results indicated that the frequency of leg movements was higher in the tinnitus group, averaging 22.09 ± 32.36, compared to 13.07 ± 20.77 in the non-tinnitus group; however, this difference was not statistically significant.

Supplementary 1 presents the characteristics and assessment results of participants with tinnitus (*n* = 65). The THI-CM score was 21.84 ± 24.04. The distribution of grades was as follows: Grade 1, 17 out of 65 (26.15%); Grade 2, 23 out of 65 (35.38%); Grade 3, 14 out of 65 (21.54%); Grade 4, 9 out of 65 (13.85%); and Grade 5, 2 out of 65 (3.08%). For air conduction pure-tone average (AC PTA) in the right ear, 35 out of 65 participants (53.85%) had hearing loss, with an average threshold of 21.77 ± 17.68 dB HL. In the left ear, 42 out of 65 participants (64.62%) had hearing loss, with an average threshold of 23.40 ± 17.02 dB HL. For bone conduction pure-tone average (BC PTA) in the right ear, 28 out of 65 participants (43.08%) had hearing loss, with an average threshold of 19.92 ± 17.88 dB HL. In the left ear, 33 out of 65 participants (50.77%) had hearing loss, with an average threshold of 21.05 ± 18.39 dB HL.

### Relationship between tinnitus-related handicap and hearing threshold, depression, and sleep disturbances

Univariate logistic regression was performed and showed that an AHI > 5 (odds ratio [OR] 2.67, *p* = 0.026)) and male sex (OR 2.49, *p* = 0.034 were associated with tinnitus (Table 2). However, in the multivariate logistic regression analysis, no significant differences in terms of these parameters were found.

**Table 2:** Logistic regression analysis of factors associated with tinnitus

Factors	Univariate				Multivariate			
	OR	(95% CI)		*p* value	OR	(95% CI)		*p* value
Male vs. Female	2.49	(1.07–	5.79)	0.034*	1.94	(0.79–	4.80)	0.150
AHI > 5 (Yes)	2.67	(1.12–	6.32)	0.026*	2.12	(0.84–	5.32)	0.111

*p < 0.05. AHI, apnoea–hypopnoea index; CI, confidence interval; OR, odds ratio

**Fig. 1:**
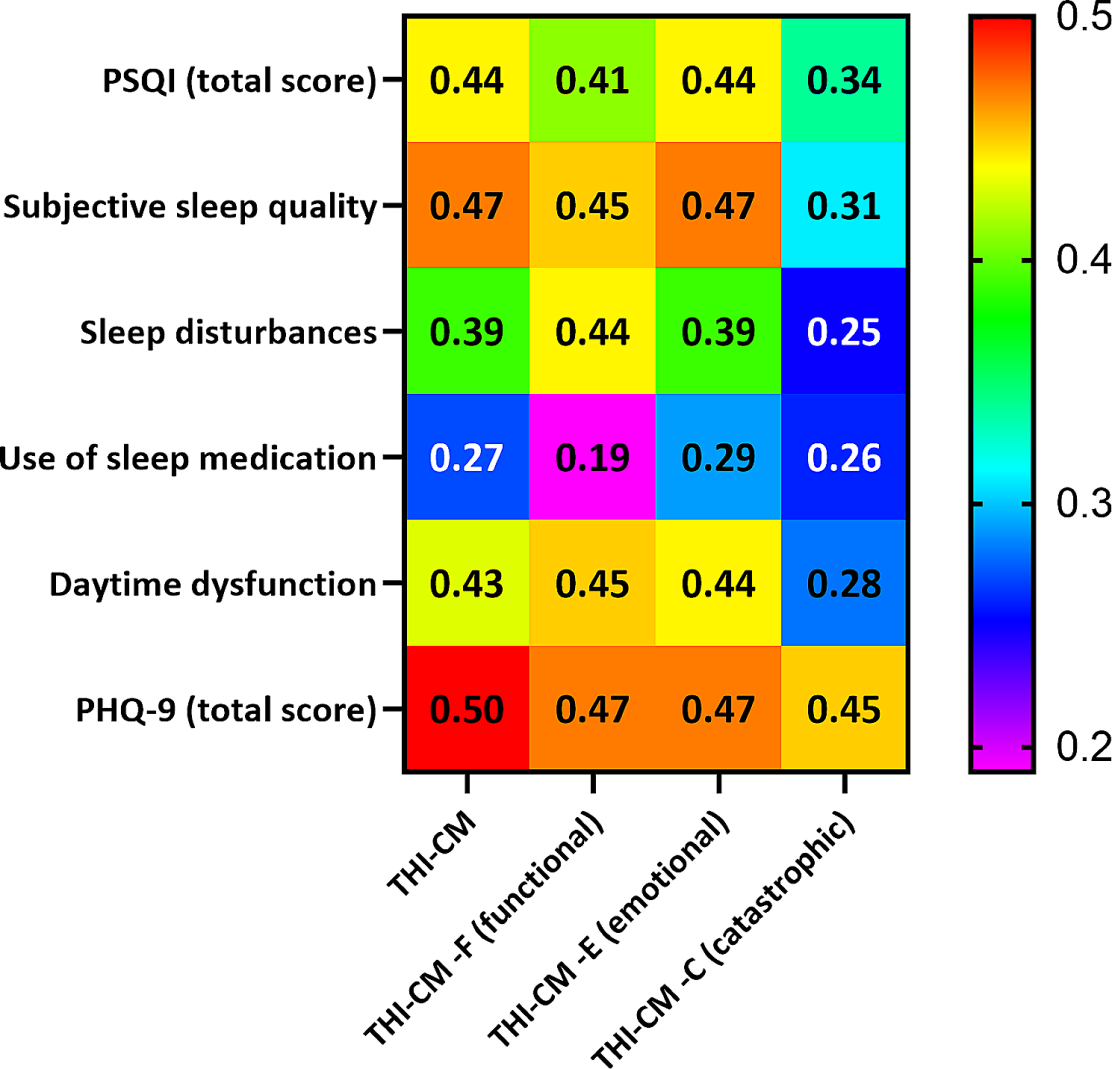
the correlations between the Chinese-Mandarin version of the Tinnitus Handicap Inventory score (THI-CM), Pittsburgh Sleep Quality Index score (PSQI) and Patient Health Questionnaire-9 score (PHQ-9)

Figure 1 shows the correlations between the THI-CM score and age, sex, PSQI, PHQ-9, polysomnographic findings, PTA, BMI, and neck and waist circumstance. A moderate positive correlation was found between the THI-CM score and total PSQI score (r_s_ =0.44, *p* < 0.001), subjective sleep quality (r_s_ =0.47, *p* < 0.001), daytime dysfunction (r_s_ =0.43, *p* < 0.001), and PHQ-9 score (r_s_ =0.50, *p* < 0.001), while a poor correlation was observed with sleep disturbance (r_s_ =0.39, *p* < 0.001). There was no correlation between the THI-CM score and variables such as hearing loss, sleep apnoea events, minimum oxygen saturation, sleep AHI, neck circumference, or waist circumference.

The participants in the tinnitus group were categorized into Grade 1, Grade 2, and Grade 3 + 5 based on their THI-CM scores for comparison (Table 3). The results revealed significant differences in PHQ-9 scores across different THI-CM severity levels (*p* < 0.001), with Grade 3 + 5 showing the highest score (9.84 ± 3.83), followed by Grade 2 (5.65 ± 4.02), and Grade 1 the lowest (4.76 ± 4.25). Similarly, when considering PHQ-9 scores ≥ 10 as indicative of depression, the proportions of depression differed across the three groups, with Grade 1 (41.18%), Grade 2 (47.83%), and Grade 3 + 5 (92.00%). PSQI total scores also exhibited a similar trend with PHQ-9 scores, indicating higher scores in more severe tinnitus groups, with Grade 3 + 5 having the highest score (12.72 ± 4.30), followed by Grade 2 (10.33 ± 3.92), and Grade 1 the lowest (7.24 ± 3.75). Among the PSQI subscales, Subjective sleep quality, Sleep disturbances, Use of sleep medication, and Daytime dysfunction were also found to be associated with THI-CM categories.

**Table 3:** THI-CM score

Characteristic	Grade 1 (*n* = 17)	Grade 2 (*n* = 23)	Grade 3–5 (*n* = 25)	*p* value
Sex				0.879
Female count (sex ratio)	5 (29.41%)	7 (30.43%)	9 (36.00%)	
Male count (sex ratio)	12 (70.59%)	16 (69.57%)	16 (64.00%)	
Age	54.89 ± 13.73	58.18 ± 7.70	52.49 ± 8.98	0.115
BMI (kg/m^2^)	25.12 ± 3.91	26.19 ± 4.17	26.19 ± 4.54	0.637
Neck circumference (cm)	36.41 ± 4.18	39.00 ± 7.47	35.83 ± 3.81	0.261
Waist circumference (cm)	87.24 ± 12.07	89.71 ± 12.77	89.15 ± 10.82	0.820
**PHQ-9 (total score)**	**4.76 ± 4.25**	**5.65 ± 4.02**	**9.84 ± 3.83**	< 0.001**
**Depression**	**7 (41.18%)**	**11 (47.83%)**	**23 (92.00%)**	0.001**
**PSQI (total score)**	**7.24 ± 3.75**	**10.33 ± 3.92**	**12.72 ± 4.30**	0.001**
**PSQI > 5**	**10 (58.82%)**	**19 (82.61%)**	**24 (96.00%)**	0.010*
**Subjective sleep quality**	**1.35 ± 0.79**	**1.70 ± 0.76**	**2.24 ± 0.60**	0.001**
Sleep latency	1.29 ± 1.16	1.26 ± 0.86	1.76 ± 0.88	0.140
Sleep duration	1.47 ± 0.80	1.57 ± 0.95	2.00 ± 0.96	0.076
Habitual sleep efficiency	0.47 ± 0.80	0.91 ± 1.12	1.24 ± 1.20	0.094
**Sleep disturbances**	**1.24 ± 0.44**	**1.74 ± 0.62**	**1.88 ± 0.67**	0.002**
**Use of sleep medication**	**0.76 ± 1.30**	**1.87 ± 1.39**	**2.00 ± 1.35**	0.017*
**Daytime dysfunction**	**0.65 ± 0.61**	**1.26 ± 1.01**	**1.60 ± 0.96**	0.006**
Apnea–Hypopnea Index	17.58 ± 17.67	21.36 ± 19.36	19.00 ± 18.79	0.832
Apnea–Hypopnea Index > 5	12 (70.59%)	17 (73.91%)	19 (76.00%)	0.926
AC PTA				
Right	19.82 ± 12.46	22.87 ± 13.91	22.08 ± 23.43	0.652
Right (hearing loss)	6 (35.29%)	10 (43.48%)	6 (24.00%)	0.359
Left	22.59 ± 11.88	27.22 ± 21.88	20.44 ± 14.69	0.363
Left (hearing loss)	8 (47.06%)	13 (56.52%)	11 (44.00%)	0.672
BC PTA				
Right	20.47 ± 11.82	19.87 ± 15.12	19.60 ± 23.43	0.243
Right (hearing loss)	7 (41.18%)	10 (43.48%)	4 (16.00%)	0.084
Left	20.06 ± 11.54	25.04 ± 25.44	18.04 ± 13.94	0.590
Left (hearing loss)	7 (41.18%)	10 (43.48%)	6 (24.00%)	0.313

Chi-Square test. Fisher exact test. Kruskal Wallis test. *p < 0.05, **p < 0.01. Continuous data are expressed as mean ± SD. Categorical data were expressed number and percentage. Hearing loss defined as any frequency threshold exceeding 20 dB HL. Pittsburgh Sleep Quality Index; PHQ-9, Patient Health Questionnaire-9; AHI, apnoea–hypopnoea index; PTA, pure-tone audiometry; AC, air conduction; BC, bone conduction; THI-CM, Chinese-Mandarin version of the Tinnitus Handicap Inventory

Table 4 conducted multinomial logistic regression analysis. Univariate analysis revealed associations between PHQ-9 (total score), PHQ-9 ≥ 10 defined as Depression, and Obstructive sleep apnea with THI-CM Grade 3–5. Further adjustment for Obstructive sleep apnea showed associations between PHQ-9 (total score) or Depression and THI-CM Grade 3–5 (OR = 1.28; OR = 8.68), with no differences observed in THI-CM Grade 2. After adjusting for sleep apnea, both original PHQ-9 (total score) and Depression defined by PHQ-9 ≥ 10 were associated with THI-CM categorical (Grade 3–5 versus Grade 1), indicating an increased risk of Grade 3–5.

When the PSQI score was > 13 and the THI-CM score was > 47, the area under the receiver operating characteristic curve was > 0.73 and > 0.75, respectively (Supplementary 2). Table 5 shows the factors associated with depression using single- and multifactor regression analyses. PSQI scores > 13 (OR 7.06, *p* = 0.018) and THI-CM scores > 47 (OR 7.43, *p* = 0.002) were significantly related to depression.

**Table 4:** Multinomial logistic regression of factors associated with **THI-CM categorical**

	Univariate								Adjusted for Obstructive sleep apnoea							
	Grade2 vs. Grade1				Grade3-5 vs. Grade1				Grade2 vs. Grade1				Grade3-5 vs. Grade1			
	OR	(95%CI)		*p* value	OR	(95%CI)		*p* value	OR	(95%CI)		*p* value	OR	(95%CI)		*p* value
Male	0.95	(0.24-	3.75)	0.944	0.74	(0.20-	2.79)	0.657								
Age	1.04	(0.97-	1.11)	0.273	0.98	(0.92-	1.04)	0.466								
BMI (kg/m^2^)	1.07	(0.91-	1.25)	0.418	1.07	(0.91-	1.25)	0.409								
Neck circumference (cm)	1.09	(0.95-	1.25)	0.204	0.97	(0.84-	1.12)	0.674								
Waist circumference (cm)	1.02	(0.96-	1.08)	0.509	1.01	(0.96-	1.07)	0.601								
PHQ-9 (total score)	1.07	(0.90-	1.28)	0.449	1.37	(1.13-	1.66)	0.001**	1.00	(0.82-	1.22)	0.996	1.28	(1.04-	1.56)	0.017*
Depression	1.31	(0.37-	4.64)	0.676	16.43	(2.89-	93.41)	0.002**	0.70	(0.15-	3.18)	0.640	8.68	(1.19-	63.32)	0.033*
Obstructive sleep apnoea	3.33	(0.78-	14.14)	0.104	16.80	(1.82-	154.89)	0.013*								
Subjective sleep quality	1.92	(0.78-	4.70)	0.155	6.15	(2.09-	18.04)	0.001**								
Sleep latency	0.96	(0.49-	1.88)	0.912	1.69	(0.86-	3.32)	0.126								
Sleep duration	1.11	(0.57-	2.17)	0.753	1.95	(0.95-	4.01)	0.069								
Habitual sleep efficiency	1.66	(0.80-	3.44)	0.172	2.14	(1.05-	4.36)	0.037*								
Sleep disturbances	4.54	(1.35-	15.22)	0.014*	6.72	(1.95-	23.22)	0.003**								
Use of sleep medication	1.80	(1.10-	2.95)	0.019*	1.93	(1.19-	3.16)	0.008**								
Daytime dysfunction	2.58	(1.08-	6.19)	0.034*	3.78	(1.55-	9.26)	0.004**								
Apnea–Hypopnea Index	1.01	(0.98-	1.05)	0.525	1.00	(0.97-	1.04)	0.799								
Apnea–Hypopnea Index > 5	1.18	(0.29-	4.78)	0.816	1.32	(0.33-	5.29)	0.696								

. *p < 0.05, **p < 0.01. Depression defined by PHQ-9 ≥ 10. PHQ-9, Patient Health Questionnaire-9

**Table 5:** Logistic regression analysis of factors associated with depression

Factors	Univariate				Multivariate			
	OR	95%CI		*p* value	OR	95%CI		*p* value
Age	0.95	(0.90-	1.01)	0.084	0.96	(0.90-	1.03)	0.279
Sex (male vs. female)	0.59	(0.19-	1.86)	0.365	0.92	(0.20-	4.30)	0.912
PSQI > 13	9.67	(2.56-	36.52)	0.001**	7.06	(1.37-	36.50)	0.020*
THI-CM > 47	12.83	(3.46-	47.65)	< 0.001**	7.43	(1.73-	32.02)	0.007**

*p < 0.05, **p < 0.01. OR, odds ratio; CI, confidence interval; **Depression, Patient Health Questionnaire-9 ≥ 10** ; PSQI, Pittsburgh Sleep Quality Index; THI-CM, Chinese-Mandarin version of the Tinnitus Handicap Inventory

## Discussion

We explored the correlation of tinnitus and tinnitus-related handicap with polysomnographic sleep characteristics, hearing, sleep quality, and depression. It is important to note that the majority of tinnitus group participants fell within the slight and mild hearing loss categories, and the average tinnitus handicap score was within the mild handicap range. Within the context of hearing and tinnitus handicap in these subjects, we found no significant correlations between tinnitus and polysomnographic sleep characteristics, sleep quality, or depression. The mild severity of tinnitus handicap scores and grouping in tinnitus participants may explain the lack of significant differences in depression, sleep quality, and polysomnographic sleep characteristics between the tinnitus and non-tinnitus groups. Tinnitus-related handicap was associated with depression and sleep quality, but not with hearing threshold or polysomnographic sleep characteristics. Multifactor analysis showed that depression moderately correlated with tinnitus-related handicap. Therefore, this study found that in the population primarily with slight or mild hearing loss and mild tinnitus handicap, depression may be a significant determinant of tinnitus-related handicap.

Previous studies have shown that the proportion of REM sleep and deep sleep is relatively lower in patients with tinnitus, and our study yielded similar results [10, 11]. Consistent with the findings of Hebert et al., the differences between the tinnitus group and the non-tinnitus group were not significant [9]. This may be attributed to the composition of the control group, as the control group in our study consisted of individuals who already had sleep disorders, which may have contributed to the lack of significance. Previous studies have utilized PSG to investigate the relationship between REM sleep and tinnitus [9–11]; however, the association between REM latency and tinnitus has not yet been confirmed. The study found no significant correlation between the severity of periodic limb movement syndrome and tinnitus [17]. However, the considerable variance in leg movement frequency, with an average of 22.09 ± 32.36/hour, suggests that clinicians should assess and consider treatment options.

Although the proportion of men and an AHI > 5 were significantly associated with tinnitus, the significance disappeared after adjusting for confounders. A meta-analysis conducted in 2022 also revealed no sex-related differences in the rate of tinnitus [1]. However, previous studies have shown that men have higher OSAS rates [27] and women with OSAS have a lower AHI and more sleep complaints [28]. This may explain why sex is not significantly associated with tinnitus after adjustment for confounders. This study’s findings diverge from previous research conclusions, as it did not observe a higher proportion of tinnitus in patients with severe obstructive sleep apnoea. Additionally, studies involving Eastern populations demonstrated a less significant association compared to those conducted in the United States or Italy [14]. In 130,788 patients with tinnitus and 108,990 controls from the Taiwan National Health Insurance Research Database, those with sleep apnoea had a higher risk of developing tinnitus than did those without sleep apnoea (adjusted OR 1.36, 95% CI = 1.16–1.60) [29]. However, based on the assumption that bias was introduced in the database, there may not necessarily be a significant correlation between tinnitus and OSAS. We found that OSAS is a common comorbidity among individuals with tinnitus. Hence, polysomnography could be more extensively employed to detect obstructive sleep apnea syndrome (OSAS) in patients with tinnitus. Clinicians should pay attention to OSAS-related symptoms and provide appropriate management, including weight loss and exercise, positive airway pressure, oral appliances, and surgical modification of the pharyngeal soft tissues or facial bones to enlarge the upper airway [30]. Moreover, patients with tinnitus often have sleep apnoea and most use sedative medications; therefore, clinical caution is necessary regarding the safety of medication use [31].

Our findings are consistent with those of previous studies showing that depression and sleep quality are related to tinnitus-related handicap [32, 33]. Our study is one of the few to use polysomnography to verify that there is no significant correlation between sleep characteristics and tinnitus [34] or tinnitus-related handicap [35]. We found that sleep quality assessed using rating scales, rather than polysomnography, correlated with tinnitus-related handicap. We inferred that depression may play a mediating role in the correlation between tinnitus and sleep quality. Additionally, serotonin depletion is believed to contribute to tinnitus because serotonin-depleting states—such as noise sensitivity, reduced REM sleep, and depression—may co-occur with tinnitus [36]. Therefore, depression plays a more influential role in tinnitus than sleep quality. As 63% of participants with tinnitus experience depression, the detection of depression and identification of suicide risk are essential when caring for individuals with tinnitus. The primary care providers in cases of tinnitus are usually general practitioners [37]. THI-CM or PSQI scores can be used to identify depression and assist in timely referral. Otherwise, patients with depression can refuse evaluation and treatment owing to stigma [38]. Referrals from an otolaryngologist or general practitioners can increase a patient’s motivation to seek psychiatric treatment.

Hearing loss contributes to tinnitus [39, 40], and hearing damage caused by OSAS-induced oxidative stress is one of the causes [41]. Hearing loss does not necessarily cause tinnitus, and individuals with tinnitus do not necessarily experience hearing loss. One possible reason for this is that hearing problems cannot be measured using appropriate tools or methods [42]. There is no conclusive evidence regarding how tinnitus affects the structural abnormalities of the brain [43]. Functional near-infrared spectroscopy studies have shown that the strength of the connection between the auditory cortex and frontotemporal, frontoparietal, temporal, occipitotemporal, and occipital cortices is enhanced during tinnitus [44]. Functional magnetic resonance imaging or other functional examinations should be used to elucidate the auditory system, limbic system, and other areas of the brain related to hearing and emotion, thereby enhancing the understanding of the relationship between depression and tinnitus [45].

Given the complex mechanism of tinnitus, a drug treatment model for tinnitus has not been established. Proven effective drugs include anticonvulsants, anxiolytics, antidepressants, antihistamines, and antiarrhythmic agents [46]. Most of these have sedative or mood-stabilizing effects. There is much overlap between the treatment modalities of tinnitus and depression, including drug therapy, cognitive behavioural therapy [47, 48], tinnitus desensitization therapy, and repetitive transcranial magnetic stimulation [49]. Collaboration between psychiatrists, neurologists and otolaryngologists is important for the future treatment of both diseases.

### Strengths and limitations

The strength of this study lies in the utilization of polysomnography and audiometric tests, enabling a more precise quantitative and qualitative evaluation of sleep and hearing variables. Apart from distinguishing between the presence and absence of tinnitus, we used a scale to understand the degree of tinnitus-related handicap. The research population originated from psychiatry and otolaryngology centres, which expands the source of participants and can better represent depression in patients with tinnitus.

However, this study also had certain limitations. The participants comprised medical centre-based patients who may have had more physiological comorbidities than average, including sleep-related breathing issues and unrecorded medical and surgical histories. These factors can cause patients to have more severe depression and poor sleep quality than the general tinnitus population or the tinnitus population not treated in hospitals. Furthermore, there is a considerable difference between the number of people in the tinnitus and non-tinnitus group. We expected a 1:1 ratio. Nevertheless, recruitment of the control group was not a smooth process, and this may result in no significant differences between the two groups. The classification of sleep disorders and depression requires further clarification. The heterogeneity of comorbidities and currently accepted treatments will affect the symptoms of participants. For example, sedative-hypnotic drugs can aggravate sleep apnoea; antidepressant drugs currently being used can improve depression and sleep, but they may also cause side effects. Cohort studies based on hospitals or national and international databases are potential future research directions. Participants underwent polysomnography only once while in hospital. There was a high proportion of participants with depression, and they may have had difficulties with emotional adjustment, which could have affected the polysomnographic results. Home sleep apnoea testing or having participants undergo polysomnography more often during a study may be future alternatives. Despite participants denying any experience of tinnitus or hearing issues, the absence of Pure Tone Audiometry (PTA) testing in the control group may have led to an oversight of undetected hearing impairments of varying degrees among the participants. Future studies should ensure that both groups undergo comprehensive hearing assessments, PTA, acoustic immittance testing, and middle ear examination.

## Conclusions

The study recruited participants primarily characterized by slight or mild hearing loss and mild tinnitus handicap, we found that depression and sleep quality detected using the PHQ-9 and PSQI were significantly correlated with tinnitus-related handicap, with depression being the main factor. Furthermore, a substantial proportion of patients with tinnitus have depression and OSAS, requiring clinical identification and treatment. Based on our findings, when patients have a PSQI score > 13 and a THI-CM score > 47, medical staff should refer them to a psychiatrist for the evaluation of depression. Larger sample sizes, long-term follow-up studies, brain imaging, and functional tests are needed to clarify the correlation between tinnitus, sleep and depression.

### Electronic supplementary material

Below is the link to the electronic supplementary material.

Supplementary Material 1

Supplementary Material 2

## Data Availability

No datasets were generated or analysed during the current study.

## Abbreviations

AHI: Apnoea–hypopnoea index
BMI: Body mass index
dB: decibel
dB-HL: decibel hearing level
OR: Odds ratio
OSAS: Obstructive sleep apnoea syndrome
PHQ-9: Patient Health Questionnaire-9
PSQI: Pittsburgh Sleep Quality Index
PTA: Pure-tone audiometry
THI-CM: Chinese-Mandarin version of the Tinnitus Handicap Inventory
REM: Rapid eye movement
