# Supplementary material for: Exploring the interplay of depression, sleep quality, and hearing in tinnitus-related handicap: insights from polysomnography and pure-tone audiometry
Source: BMC Psychiatry. 2024 Jun 19;24:459. doi: 10.1186/s12888-024-05912-y (PMC11186200; doi:10.1186/s12888-024-05912-y)
Supplement: Supplementary file 1 — Supplementary Material 1 [file 12888_2024_5912_MOESM1_ESM.docx]

| Supplementary table 1. The characteristics and assessments results of the participants with tinnitus (n=65) | | |
| --- | --- | --- |
| Characteristic | Total (n=65) | |
| THI-CM | 21.84 | ±24.04 |
| Severity |  |  |
| Grade 1 | 17 | (26.15%) |
| Grade 2 | 23 | (35.38%) |
| Grade 3 | 14 | (21.54%) |
| Grade 4 | 9 | (13.85%) |
| Grade 5 | 2 | (3.08%) |
| THI-CM -F (functional) | 9.00 | ±10.24 |
| THI-CM -E (emotional) | 6.68 | ±8.97 |
| THI-CM -C (catastrophic) | 6.16 | ±5.99 |
| PTA |  |  |
| AC PTA - Right | 21.77 | ±17.68 |
| AC PTA - Right (hearing loss) | 35 | (53.85%) |
| AC PTA - Right |  |  |
| Normal | 30 | (46.15%) |
| Slight | 18 | (27.69%) |
| Mild | 12 | (18.46%) |
| Moderate | 1 | (1.54%) |
| Moderately severe | 3 | (4.62%) |
| Severe | 0 | (0.00%) |
| Profound | 1 | (1.54%) |
| AC PTA - Left | 23.40 | ±17.02 |
| AC PTA - Left (hearing loss) | 42 | (64.62%) |
| AC PTA - Left |  |  |
| Normal | 23 | (35.38%) |
| Slight | 25 | (38.46%) |
| Mild | 11 | (16.92%) |
| Moderate | 2 | (3.08%) |
| Moderately severe | 2 | (3.08%) |
| Severe | 1 | (1.54%) |
| Profound | 1 | (1.54%) |
| BC PTA - Right | 19.92 | ±17.88 |
| BC PTA - Right (hearing loss) | 28 | (43.08%) |
| BC PTA - Right |  |  |
| Normal | 37 | (56.92%) |
| Slight | 16 | (24.62%) |
| Mild | 7 | (10.77%) |
| Moderate | 1 | (1.54%) |
| Moderately severe | 3 | (4.62%) |
| Severe | 0 | (0.00%) |
| Profound | 1 | (1.54%) |
| BC PTA - Left | 21.05 | ±18.39 |
| BC PTA - Left (hearing loss) | 33 | (50.77%) |
| BC PTA - Left |  |  |
| Normal | 32 | (49.23%) |
| Slight | 19 | (29.23%) |
| Mild | 8 | (12.31%) |
| Moderate | 3 | (4.62%) |
| Moderately severe | 2 | (3.08%) |
| Severe | 0 | (0.00%) |
| Profound | 1 | (1.54%) |
| Continuous data are expressed as mean±SD. THI-CM, Chinese-Mandarin version of the Tinnitus Handicap Inventory; PTA, pure-tone audiometry; AC, air conduction; BC, bone conduction | | |
