# Supplementary material for: Exploring the interplay of depression, sleep quality, and hearing in tinnitus-related handicap: insights from polysomnography and pure-tone audiometry
Source: BMC Psychiatry. 2024 Jun 19;24:459. doi: 10.1186/s12888-024-05912-y (PMC11186200; doi:10.1186/s12888-024-05912-y)
Supplement: Supplementary file 2 — Supplementary Material 2 [file 12888_2024_5912_MOESM2_ESM.docx]

| \| Supplementary table 2. Receiver operating characteristic curve of depression (n=65) \| \| \| \| \| \| \| --- \| --- \| --- \| --- \| --- \| --- \| \| Variable \| AUC \| Cut-off point \| Sensitivity \| Specificity \| Accuracy \| \| PSQI \| 0.73 \| >13 \| 52.94 \| 89.58 \| 80.00 \| \| THI-CM \| 0.75 \| >47 \| 64.71 \| 87.50 \| 81.54 \|   AUC, Area under the curve; PSQI, Pittsburgh Sleep Quality Index; THI-CM, Chinese-Mandarin version of the Tinnitus Handicap Inventory |
| --- | --- | --- | --- | --- | --- | --- | --- | --- | --- | --- | --- | --- | --- | --- | --- | --- | --- | --- | --- | --- | --- | --- | --- | --- |
